# Ghrelin Increases Beta-Catenin Level through Protein Kinase A Activation and Regulates OPG Expression in Rat Primary Osteoblasts

**DOI:** 10.1155/2015/547473

**Published:** 2015-03-17

**Authors:** Emanuela Mrak, Lavinia Casati, Francesca Pagani, Alessandro Rubinacci, Guido Zarattini, Valeria Sibilia

**Affiliations:** ^1^Department of Medical Biotechnology and Translational Medicine, Medical Pharmacology Unit, Università degli Studi di Milano, Via Vanvitelli 32, 20129 Milano, Italy; ^2^Bone Metabolism Unit, Scientific Institute San Raffaele, Via Olgettina 60, 20132 Milano, Italy; ^3^Department of Medical and Surgical Specialties, Radiological Sciences and Public Health, University of Brescia, Viale Europa 11, 25123 Brescia, Italy

## Abstract

Ghrelin, by binding growth hormone secretagogue receptor (GHS-R), promotes osteoblast proliferation but the signaling mechanism of GHS-R on these cells remains unclear. Since canonical Wnt/*β*-catenin pathway is critically associated with bone homeostasis, we investigated its involvement in mediating ghrelin effects in osteoblasts and in osteoblast-osteoclast cross talk. Ghrelin (10^−10^M) significantly increased *β*-catenin levels in rat osteoblasts (rOB). This stimulatory action on *β*-catenin involves a specific interaction with GHS-R1a, as it is prevented by the selective GHS-R1a antagonist, D-Lys^3^-GHRP-6 (10^−7^M). The effect of ghrelin on *β*-catenin involves the phosphorylation and inactivation of GSK-3*β* via protein kinase A (PKA). Inhibition of PKA activity reduces the facilitatory action of ghrelin on *β*-catenin stabilization. Ghrelin treatment of rOB significantly increases the expression of osteoprotegerin (OPG), which plays an important role in the regulation of osteoclastogenesis, and this effect is blocked by D-Lys^3^-GHRP-6. Furthermore, ghrelin reduced RANKL/OPG ratio thus contrasting osteoclastogenesis. Accordingly, conditioned media from rOB treated with ghrelin decreased the number of multinucleated TRAcP+ cells as compared with the conditioned media from untreated-control rOB. Our data suggest new roles for ghrelin in modulating bone homeostasis via a specific interaction with GHSR-1a in osteoblasts with subsequent enhancement of both *β*-catenin levels and OPG expression.

## 1. Introduction

Accumulating evidence, including in vitro and in vivo studies in animal models as well as observational studies in humans, indicate a role for ghrelin in the control of bone cell activities [1–4].

Ghrelin, an endogenous ligand of the GH secretagogue receptor (GHS-R), and its receptor GHS-R1a are expressed in several osteoblasts and osteoblastic cell lines. Ghrelin directly promotes both osteoblast proliferation and differentiation [5–8] and inhibits apoptosis of MC3T3-E1 cells [6, 9]. Moreover, recent data suggest that GHS-R1a expression is subjected to an epigenetic regulation through DNA methylation and histone modification. Changes in the epigenetic status of the cell might therefore play an important role in determining sensitivity to ghrelin [10].

In addition to a well characterized anabolic action, the data regarding the effects of ghrelin on osteoclastogenesis are contradictory. Costa et al. [8] have shown that ghrelin increases the bone resorption activity of rat osteoclasts but did not modify osteoclast differentiation in a murine bone marrow assay or bone resorption in ex vivo calvarial culture. More recently, van der Velde et al. [11] found that ghrelin has dual actions in osteoclasts since it inhibits osteoclast progenitors locally and exerts an age-dependent stimulatory action on osteoclastogenesis via a systemic pathway.

In vivo studies showed that intraperitoneal [5] as well as central administration [12] of ghrelin increases bone mineral density and mass through a mechanism independent of GH-IGF1 axis and appetite regulation, respectively. Consistently, synthetic GH secretagogues increase bone mineral density [13] and prevent gonadectomy-induced bone loss in the rat [14, 15]. Data from clinical studies are limited but indicate a positive association between circulating ghrelin levels and bone mineral density in women [16, 17] and elderly men [18].

Despite convincing evidence that ghrelin has positive effects on bone metabolism, the signaling mechanism and the specific involvement of GHS-R1a in osteoblast are still controversial [19] and remain unclear. Since the canonical Wnt/*β*-catenin pathway is critically associated with the entire osteoblast life cycle, that is, commitment, proliferation, apoptosis, and function, we hypothesized that ghrelin exerts its anabolic action in bone through canonical Wnt signaling and *β*-catenin stabilization. This hypothesis is supported by the observation that in rat cortical neuron cells ghrelin exerts specific, receptor mediated antiapoptotic effects by activating ERK1/2 pathway and by stabilizing *β*-catenin via PI3K/Akt-mediated inactivation of glycogen synthase kinase (GSK-3*β*) [20].

Wnt canonical signaling pathway relies on the cytosolic stabilization of *β*-catenin, which is a 130 amino acid protein that, in the absence of Wnt proteins, is phosphorylated by GSK-3*β* and degraded through the proteosomal machinery. When not degraded, stable *β*-catenin is accumulated in the cytoplasm and upon reaching a certain concentration level is translocated into the nucleus to stimulate gene transcription [21].

To test our hypothesis, we first examined the effects of increasing concentrations of ghrelin on *β*-catenin intracellular levels in primary rat osteoblasts. Then, we characterized the receptor involved in the stimulatory action of ghrelin on *β*-catenin and examined the effects of ghrelin on the expression of osteoprotegerin (OPG) a target gene of *β*-catenin that plays an important role in osteoblasts and osteoclasts cross talk. Furthermore, we evaluated whether or not ghrelin, by modulating the OPG/RANKL/RANK system, could influence osteoclastogenesis in cultured rat osteoclast precursors cells.

## 2. Materials and Methods

### 2.1. Drugs

Ghrelin was synthesized by conventional solid phase synthesis and purified to at least 98% purity by HPLC by Neosystem (Strasburg, France). D-Lys^3^-GHRP-6 was purchased from Bachem AG (Dubendorf, Switzerland). H89 was from Sigma, Milan, Italy. Rat macrophage colony-stimulatory factor (M-CSF) and rat RANKL were from Peprotech, Rocky Hill, NJ.

### 2.2. Rat Osteoblast-Like Cells

Parietal and frontal calvariae (4 per animal) were explanted aseptically from 3-day-old Sprague Dawley rats. After removing the periosteum, the calvariae were cut in small pieces and placed in Joklik's modified MEM (Sigma, Milan, Italy) serum-free medium containing 0.5 mg/mL type IV collagenase (Sigma, Milan, Italy) for 20 minutes at 37°C with rotation. Digestion was stopped by the addition of Dulbecco's medium (DMEM, Euroclone, Milan, Italy) containing 10% foetal bovine serum (FBS, Euroclone). Calvariae obtained from three animals were plated in 75 cm^2^ flask and allowed to grow to confluence in DMEM containing 10% FBS, 100 U/mL penicillin, 100 mg/mL streptomycin, and 0.25 mg/mL amphotericin B; the culture medium was changed every 2-3 days. Cells reached confluence after approximately one week. The cell population was tested for alkaline phosphatase production after PTH 10^−8 ^M treatment for 48 h to ensure that the cells were endowed with osteoblast characteristics. Cells were all used at first passage to reduce the possibility of phenotype changes.

### 2.3. Cell Viability Assay

Cells were plated at the density of 3 × 10^3^ cells/well in a 96-well culture plate. After treatment, cells were washed with phosphate buffered saline (PBS, Euroclone, Italy) and incubated at 37°C with 0.5 mg/mL 3-(4,5-dimethyl-2-thiazolyl)-2,5-diphenyltetrazoliumbromide (MTT, Sigma-Aldrich Chemical, Italy) for 3 h. The conversion of the tetrazolium salt MTT to a colored formazan was used to assess cell viability. After the supernatant was removed, dimethyl sulfoxide was added to each well and the absorbance was recorded by a microplate spectrophotometer (WALLAC) at 550 nm.

Cell phenotypic observations were made using Olympus TH4-200 inverted phase-contrast microscope, fitted with a digital camera Olympus C-4040 zoom to record any change during treatment.

### 2.4. Western Blot Analysis

Confluent cells plated in 6-well multiwell plates were treated with ghrelin 10^−11^–10^−7 ^M for 15, 30, 60, 90, and 120 min; the GHSR1a receptor antagonist, D-Lys^3^-GHRP-6 (10^−7 ^M), was added 30 min before ghrelin 10^−10 ^M; incubation with 2 × 10^−6^ M H89, an inhibitor of PKA activity, was performed 30 min before ghrelin 10^−10 ^M.

After removing the medium, adherent cells were gently scraped into 75 *μ*L RIPA buffer (1% triton X100, 0.5% Sodium deoxycholate, 0.1% Sodium Dodecyl Sulphate) per well. Two wells were collected for each treatment. Total protein concentration was determined by BCA assay (Pierce, Rockford, IL). Thirty micrograms of total protein extract was mixed with the appropriate volume of Laemmli's sample loading buffer, heated at 100°C for 5 min and loaded onto 10% SDS polyacrylamide gels. Western blots were performed using a specific antibody against rat *β*-catenin diluted 1 : 5000 in 5% milk in Tris–HCl with 0.1% Tween-20 (TBST); rat P-GSK3*β* (1 : 2000 5% milk in TBST); rat total GSK3*β* (1 : 2000 5% milk in TBST) (Cell Signaling Technology, Boston, MA) or against rat *β*-actin (1 : 2000 5% milk in TBST) (Santa Cruz Biotechnology, Inc., Heidelberg, Germany). Nuclear fractions were isolated from untreated cells or cells treated with ghrelin (10^−10 ^M) alone or in association with D-Lys^3^-GHRP-6 (10^−7 ^M) by the Qproteome Cell Compartment Assay (Qiagen SpA, Milan, Italy) that allows selectively isolating proteins in different cell compartments by sequential addition of different extraction buffers to a cell pellet. Scion Image software (Scion Corp., Frederick, MD) was used to quantify the Western blots. *β*-Actin or Tot-GSK3*β* was used as endogenous controls to avoid incorrect estimations of the signal from the protein of interest. The intensity of the treated samples was normalized to that of the untreated samples (set as 1) and presented as a ratio of untreated controls.

### 2.5. Real-Time PCR

Osteoprotegerin (OPG), *β*-catenin, receptor activator of NFkB ligand (RANKL), and Dickkopf 1 (DKK1) mRNA relative expression were evaluated by real-time PCR after exposing rOB at confluence to ghrelin 10^−11 ^M–10^−7 ^M for 1, 4, and 24 h in serum-free medium. In order to assess if the effect of ghrelin on OPG gene expression was receptor mediated, cells were pretreated with D-Lys^3^-GHRP-6 10^−7 ^M 30 min before adding ghrelin. Total RNA was extracted using TRIzol according to the manufacturer's instructions (Invitrogen Life Technology, Inc., Paisley, UK). RNA pellet concentrations were assessed spectrophotometrically using Epoch Spectrophotometer System (Biotek) (OD260/280). Two micrograms of total RNA were retrotranscribed in a total volume of 25 *μ*L using an oligodT primer (0.5 mM), 200 U of M-MLV reverse transcriptase, deoxynucleotides (0.5 mM), M-MLV reaction Buffer 1x, and rRNasin ribonuclease inhibitor 1 U/mL (Promega Corporation, Madison, WI). Relative quantification of OPG *β*-catenin, RANKL, and DKK1 was performed on an ABI PRISM 7900 sequence detector (Applied Biosystems, Foster City, CA) using 20 ng cDNA of the RT-PCR solution in a final volume of 25 *μ*L. The primer-probe sets were purchased as assay-on-demand for gene expression from Applied Biosystems. Real-time PCR was performed with FAM-labelled specific probes for OPG, *β*-catenin, RANKL, and DKK1 detection and VIC-labelled probes for the detection of the housekeeping gene glyceraldehyde-3-phosphate dehydrogenase (GAPDH), used as an endogenous control. Real-time PCR was run according to the following protocol: an initial step of 2 min at 50°C and 10 min at 95°C followed by 40 cycles of 15 sec at 95°C and 1 min at 60°C. mRNA levels were quantified using the comparative threshold-cycle (Ct) method.

### 2.6. Osteoclastogenesis Assay

Osteoclast precursors cells were obtained from 3-day-old rat pups long bones. Bone marrow was flushed out with DMEM and the cell suspension was pushed through needles of decreasing size (19 G–25 G) until homogenously resuspended. Cells were then centrifuged and plated in petri dishes in DMEM supplemented with 10% FBS, 100 U/mL penicillin, 100 mg/mL streptomycin, and 0.25 mg/mL amphotericin B in the presence of M-CSF (100 ng/mL) for 48 h. Nonadherent erythrocytes were removed while adherent bone marrow cells were washed with PBS resuspended in culture medium and plated on 96-well plates (10^4^ cells/well) with M-CSF (25 ng/mL) and RANK-L (50 ng/mL) for osteoclast generation in presence of 50% of conditioning medium derived from rOB treated with ghrelin 10^−10 ^M for 24 h, 48 h, or untreated. Half of the media was changed every day and cytokines were added freshly. After 5 days of culture cells were fixed with 4% paraformaldehyde in PBS for 10 min and osteoclast formation was assessed by tartrate resistant acid phosphatase (TRAcP) staining. Briefly, fixed cells were incubated with naphthol-ASBI-phosphate, pararosanilin, and tartrate in acetate buffer (30 *μ*M) at 37°C for 45 min. TRAcP positive cells with three or more nuclei were considered to be osteoclasts.

### 2.7. Statistical Analysis

Statistical analysis was performed with a statistic package (GraphPad Prism 5, GraphPad Software San Diego, CA, USA). All data are represented as the mean ± SEM. Differences between groups were assessed by one-way analysis of variance (ANOVA) for nonparametric data (Kruskal-Wallis test) followed by a multiple comparison test (Dunn's test) and by Mann-Whitney *U* test when comparing two groups only. A probability of *P* < 0.05 was considered to be significant.

## 3. Results

**Figure 1:**
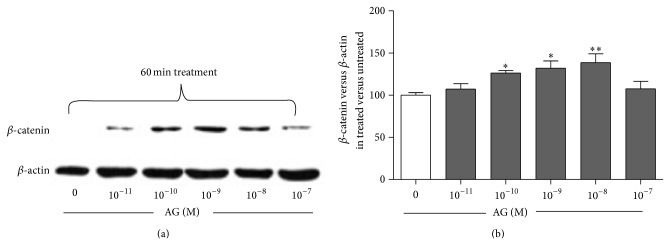
Representative Western blot (a) and quantification (b) of the effect of increasing concentrations of ghrelin (AG, 10^−11^–10^−7 ^M, 1 h treatment) on *β*-catenin protein level in whole cell lysates of rat osteoblast-like cells. *β*-catenin levels are normalized on *β*-actin protein levels and results are expressed as the ratio of *β*-catenin amount measured in treated cells to the amount obtained in untreated cells. Data are the mean ± SEM of five experiments ^*^ *P* < 0.05, ^**^ *P* < 0.01 versus untreated cells.

To examine the effects of ghrelin on *β*-catenin levels, primary rat osteoblast-like cells were treated for 60 min with increasing ghrelin concentrations. Using Western blot analysis, we have shown that ghrelin significantly increased *β*-catenin levels starting from 10^−10 ^M to 10^−8 ^M (Figure 1). Although the observed increment was small, it was dose and time dependent, thus indicating its specificity.

**Figure 2:**
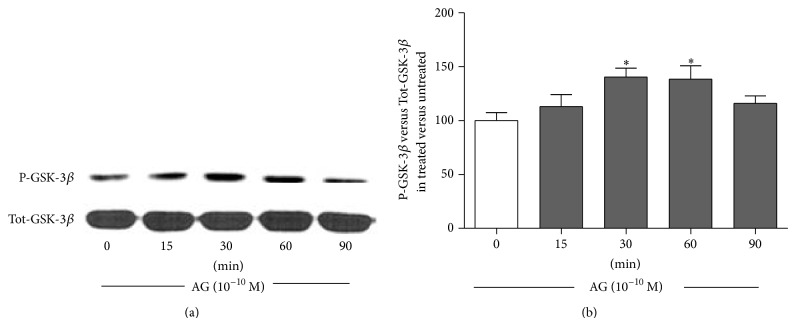
Representative Western blot (a) and quantification (b) of timecourse experiments performed on rat osteoblast-like cells treated with ghrelin (AG, 10^−10 ^M) for 15, 30, 60, and 90 min. Phosphorylated-GSK-3*β* (P-GSK-3*β*) levels were measured in whole cell lysates and normalized to total GSK-3*β* levels (Tot-GSK3*β*). Results are shown as ratio of P-GSK3*β* measured in treated versus untreated cells. Data are the mean ± SEM of 5 experiments ^*^ *P* < 0.05, versus untreated cells.

The stimulatory action of ghrelin on *β*-catenin involves GSK-3*β* phosphorylation and inactivation as shown by the significant increase of phosphorylated GSK-3*β* induced by ghrelin (10^−10 ^M) starting from 30 min of incubation (Figure 2).

**Figure 3:**
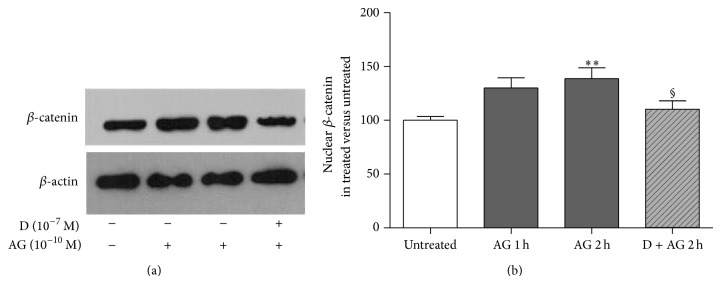
Representative Western blot (a) and quantification (b) of *β*-catenin levels in the enriched nuclear fraction of rat osteoblast-like cells exposed to ghrelin (AG, 10^−10 ^M for 1 and 2 h) alone or together with D-Lys^3^-GHRP-6 (D, 10^−7 ^M 30 min before AG), a GHSR-1a receptor antagonist. *β*-catenin levels are normalized on *β*-actin protein levels and results are expressed as the ratio of *β*-catenin amount measured in treated cells to the amount obtained in untreated cells. Data are the mean ± SEM of five experiments ^**^ *P* < 0.01 versus untreated; ^§^ *P* < 0.05 versus 2 h AG treated cells.

In order to study whether the observed *β*-catenin stabilization induced by ghrelin involves a specific interaction with its GHS-R1a, primary rat osteoblast-like cells were treated for 30 min with the selective GHS-R1a antagonist, D-Lys^3^-GHRP-6 (10^−7 ^M), and then challenged with ghrelin (10^−10 ^M). *β*-catenin levels were measured in both the cytosolic and nuclear compartments after fractioning of the whole cell lysate. Pretreatment with D-Lys^3^-GHRP-6 abolished both ghrelin-induced cytoplasm *β*-catenin stabilization and *β*-catenin nuclear migration (Figure 3), indicating that the effects of ghrelin on *β*-catenin are due to a specific interaction of the peptide with GHS-R1a.

**Figure 4:**
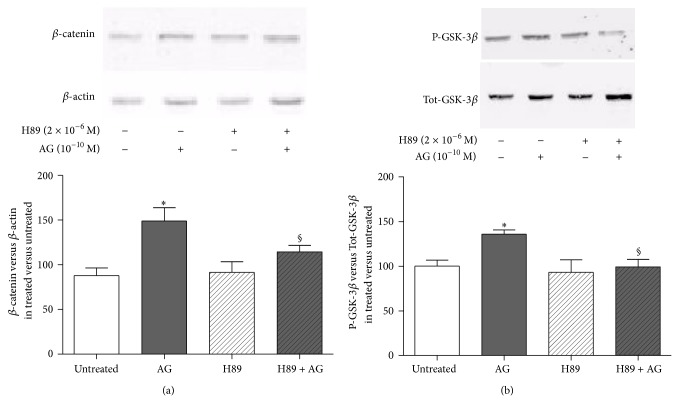
Effect of the pretreatment with the PKA inhibitor H89 (2 × 10^−6^ M, 30 min before) on (a) *β*-catenin and (b) phosphorylated-GSK3*β* (P-GSK3*β*) levels measured in whole cell lysates by Western blot on rat osteoblast-like cells (rOB) treated with ghrelin (AG, 10^−10 ^M for 1 h). *β*-catenin levels were normalized to *β*-actin levels; P-GSK3*β* was normalized to total GSK3*β* levels (Tot-GSK3*β*). Results are expressed as ratio of *β*-catenin or P-GSK3*β* measured in treated cells versus untreated cells.* Insets*: typical gels obtained from rOB cell lysates after treatment with ghrelin alone or together with H89. Data are the mean ± SEM of 5 experiments ^*^ *P* < 0.05 versus untreated cells; ^§^ *P* < 0.05 versus AG treated cells.

We then explored the intracellular signaling pathways involved in the stimulatory action of ghrelin on *β*-catenin levels following its binding to GHS-R1a. Our data demonstrate that 10^−10 ^M ghrelin activates cAMP/PKA pathway since a PKA inhibitor, H89, reduced both ghrelin-induced *β*-catenin stabilization (Figure 4(a)) and GSK-3*β* phosphorylation (Figure 4(b)).

**Figure 5:**
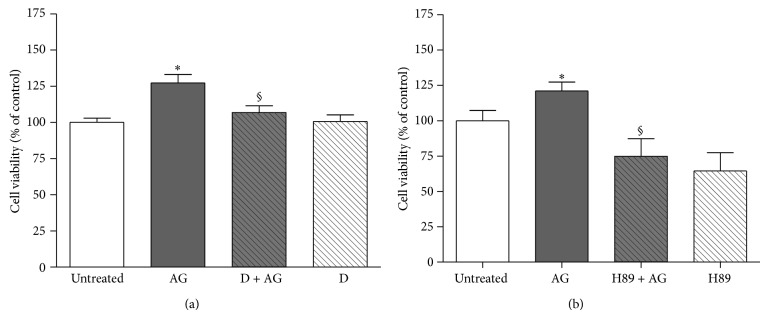
Effect of pretreatment with (a) D-Lys^3^-GHRP-6 (D, 10^−7 ^M, 30 min before AG) or (b) the PKA inhibitor H89 (2 × 10^−6^ M, 30 min before AG) on the stimulatory action of ghrelin (AG, 10^−10 ^M) on rat osteoblast-like cells (rOB) viability. Cell viability was measured by MTT assay. Data are expressed as the percentage relative to control and are the means ± SEM of four replicates within a single experiment that was repeated at least three times. ^*^ *P* < 0.05 versus untreated cells; °*P* < 0.05 versus AG.

To further confirm that ghrelin exerts its anabolic action by interacting with GHS-R1a via PKA activation, osteoblast viability was assessed after pretreatment with D-Lys^3^-GHRP-6 (Figure 5(a)) and after inhibition of PKA activity by H89 (Figure 5(b)). As shown, ghrelin increases significantly cell viability whereas both pretreatments nullified it.

**Figure 6:**
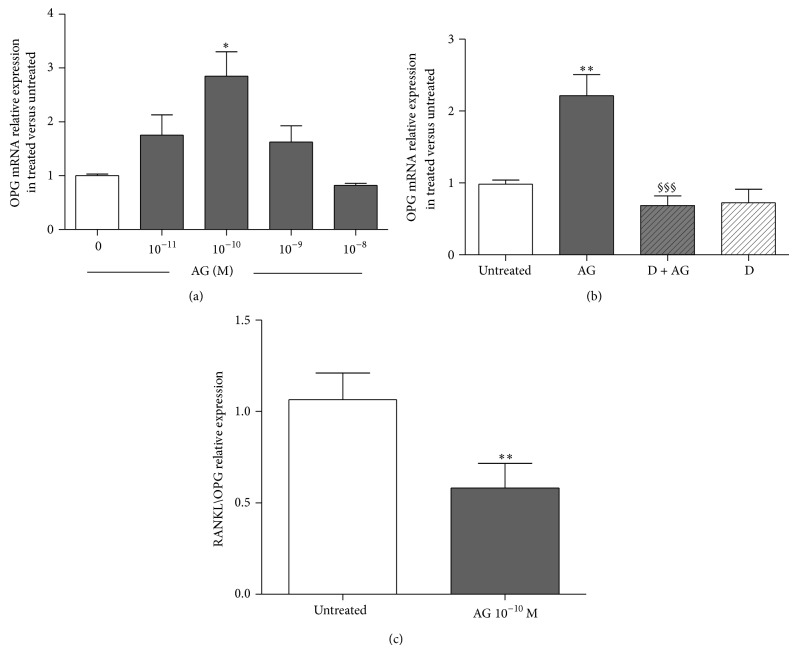
Effect of increasing concentrations of ghrelin (AG, 10^−11 ^M–10^−7 ^M) on osteoprotegerin (OPG) expression measured by real-time PCR at 24 h after AG treatment in primary cultures of rat osteoblast-like cells (a). Effect of pretreatment (30 min before) with the GHSR-1a antagonist D-Lys^3^-GHRP-6 (D, 10^−7 ^M) on the stimulatory action of AG (10^−10 ^M for 24 h) on OPG expression (b). Ratio of RANKL/OPG relative expression measured in untreated and AG 10^−10 ^M treated rat osteoblasts after 24 h (c). OPG and RANKL expression were normalized to that of GAPDH and expressed as 2^−ΔΔCt^. Three replicates were performed for each experimental point and experiments were repeated three times. ^*^ *P* < 0.05; ^**^ *P* < 0.01 versus untreated cells. ^§§§^ *P* < 0.001 versus AG treated cells.

Considering that OPG is a target gene of *β*-catenin, we examined the effects of increasing ghrelin concentrations on the expression of OPG, *β*-catenin, and DKK1 measured by real-time PCR. Primary rat osteoblast-like cells treated for 24 h with ghrelin (10^−10 ^M) showed a significant increase in the expression of OPG. This ghrelin action involves an interaction with GHS-R1a since it was removed by pretreatment with D-Lys^3^-GHRP-6 (Figures 6(a) and 6(b)). We next examined the effects of increasing ghrelin concentration on RANKL expression. Ghrelin had no significant effect on RANKL mRNA but significantly reduced RANKL/OPG ratio at 10^−10 ^M (Figure 6(c)). No significant effect on *β*-catenin and DKK1 expressions at 1, 4, and 24 h of exposure to different doses of ghrelin (from 10^−11 ^M to 10^−7 ^M) was found (data not shown).

**Figure 7:**
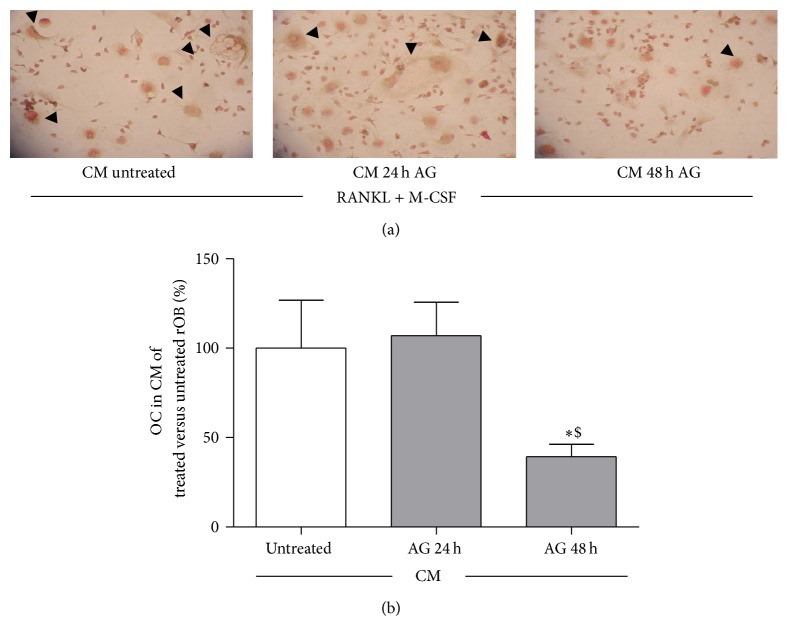
Effect of conditioned media (CM) obtained from rat osteoblast-like cells (rOB) treated without or with ghrelin (AG, 10^−10 ^M for 24 or 48 h) on osteoclast formation, in presence of 50 ng/mL RANKL and 25 ng/mL M-CSF.* Black arrows*: multinucleated tartrate-resistant acid phosphatase positive (TRAP+) cells (a). The percentage of TRAP+ multinucleated cells (≥3 nuclei) formed after 7 days culture with conditioned media obtained from untreated and treated (AG, 10^−10 ^M for 24 or 48) rOB (b). ^*^ *P* < 0.05 versus CM of untreated cells; ^$^ *P* < 0.05 versus CM of rOB treated for 24 h with AG.

To test the involvement of ghrelin in the regulation of osteoclastogenesis, due to its stimulatory action on OPG expression, we examined the effects of conditioned media from rat osteoblasts treated with ghrelin, on osteoclast precursors. Osteoclast precursors cells were obtained by bone marrow of long bones and cultured for seven days in conditioned media obtained from rOB treated with or without Ghrelin 10^−10 ^M for 24 and 48 h, in the presence of M-CSF (25 ng/mL) and of a subeffective dose of RANKL (50 ng/mL). The presence of osteoclast-like cells was evaluated by counting the number of multinucleated TRAcP positive cells per well. The number of osteoclasts was significantly reduced as compared with that detected incubating osteoclast precursors with medium from untreated-control osteoblasts, when bone marrow precursors were exposed to conditioned media from 48 h ghrelin- (10^−10 ^M) treated rOB, (Figure 7).

## 4. Discussion

In the present study we have shown that the anabolic action of ghrelin involves an increase in the *β*-catenin levels in primary rat osteoblastic cells. This effect is mediated by GHS-R1a as its specific antagonist, D-Lys^3^-GHRP-6, blocks it. Our findings indicate that the effect of ghrelin on *β*-catenin accumulation is due to inhibition of GSK-3*β* activation via cAMP/PKA pathway since the inhibition of the enzyme activity by H89 reduces ghrelin activity. Consistently with the activation of Wnt signaling, ghrelin also increases the expression of OPG and decreases RANKL/OPG ratio leading to reduced osteoblast-related osteoclast formation.

Several studies have shown anabolic action of ghrelin on bone [5–7]; however the mechanisms for this effect remain to be fully elucidated. One possible mechanism includes the secretagogue action of ghrelin on growth hormone [22]. However, it is unlikely that the effects of ghrelin on bone are exclusively mediated through a GH/IGF-1 axis since ghrelin administration increases bone mass in genetically GH-deficient rats [5].

Conversely, our study was based on the hypothesis that ghrelin, acting on osteoblasts, could exert a direct anabolic action through an autocrine/paracrine way that involves Wnt/*β*-catenin canonical pathway: a critical signaling controlling osteoblasts at different levels, that is, commitment, proliferation, apoptosis and function [23]. In the absence of active Wnt ligands, *β*-catenin is bound to the scaffold proteins Axin and adenomatous polyposis coli (APC) and constitutively phosphorylated via interaction with casein kinase I and GSK3-*β*. The presence of Wnt ligands inhibits the kinase activity of GSK-3*β* leading to *β*-catenin accumulation in the cytoplasm followed by its translocation into the nucleus where it interacts with T cell factor/lymphoid enhancer family members to induce the transcription of key osteoblastic genes [24]. Canonical Wnt signaling is crucial for osteoblastogenesis and bone formation since loss or gain of function of *β*-catenin is associated with a decrease or an increase of bone mass, respectively [25]. Here we showed that ghrelin increased *β*-catenin cytoplasmic levels leading to the nuclear translocation of the protein. The effect was obtained under physiological dosages of ghrelin to show its fine regulation of bone cells; its magnitude was in agreement with data recently observed in other systems [26]; it was dose and time dependent and it was consistent with the inhibition of GSK-3*β*: a critical element of *β*-catenin activation.

The observation that ghrelin increases GSK-3*β* phosphorylation (Figure 2(a)) suggests that ghrelin promotes *β*-catenin stabilization by downregulating its inhibitory pathway. Our data are in agreement with previous studies showing that the activation of phosphorylated GSK-3*β*/*β*-catenin activity is involved in the beneficial effects of ghrelin against hypoxia-induced pulmonary hypertension in the rat [27] and ischemic neural injury [20, 28]. In our study, the direct effect of PKA on GSK-3*β* was documented since H89 nullified ghrelin effect on GSK-3*β* activity. The direct effect of PKA on *β*-catenin phosphorylation and degradation seems to be ruled out on the basis of the present results showing that pretreatment with H89 increased *β*-catenin level instead of reducing it.

Moreover, our results sustain the emerging view that several classes of hormones and peptides activate *β*-catenin signaling through PKA dependent inhibition of GSK-3*β* as was demonstrated for PTH [29] and calcitonin gene related peptide [30]. The activation of Wnt signaling through GSK-3*β* inhibition and* vice versa *is in fact becoming a critical element for the full understanding of the anabolic role of Wnt [31]. Besides the possible involvement of a receptor other than GHS-R1a in ghrelin dependent bone metabolism regulation [19], our study has demonstrated the importance of GHS-R1a in the activation of the Wnt signaling in osteoblast.

Considering that GHS-R1a has been identified in osteoblasts [1, 7] and that at the moment the only acknowledged ghrelin receptor is GHS-R1a, we examined the involvement of this receptor in the stimulatory action of ghrelin on *β*-catenin levels. GHS-R1a involvement was proven by the fact that the specific GHS-R1a antagonist, D-Lys^3^-GHRP-6, abolished the stimulatory action of ghrelin on *β*-catenin, indicating that this ghrelin effect is GHS-R1a- mediated. These results fit also well with the report that GHS-R1a is involved in the stimulatory action of ghrelin on osteoblast proliferation [5] and with the present results showing that D-Lys^3^-GHRP-6 reduced the stimulatory action of ghrelin on osteoblast viability. Furthermore, upregulation of phosphorylation of GSK-*β*/*β*-catenin signaling in the presence of GHS-R1a protected neonatal rats from hypoxia-induced pulmonary hypertension [27].

Stimulation of ghrelin receptor leads to activation of multiple downstream signaling.

Kim and colleagues [6] have shown that ghrelin promotes osteoblast proliferation and differentiation through MAPK/ERK and PI3K/AKT signaling pathways, but cAMP-mediated PKA pathway was not examined. It is likely that the effects of ghrelin on *β*-catenin accumulation involve PKA activation since inhibition of the enzyme activity by H89 abrogated the stimulatory action of ghrelin on *β*-catenin levels. Any potential bias due to the effect of H89 on cell differentiation was controlled by the short term (1 hour) exposure of the cultures. It remains to be clarified whether ghrelin-induced *β*-catenin stabilization via cAMP-dependent protein kinase activation involves only the inhibition of GSK-3*β*.

A number of recent reports showed that the phosphorylation of *β*-catenin on ser552 by PKA enhances the transcriptional ability of *β*-catenin independent of the phosphorylation of *β*-catenin in the N-terminal domain at canonical sites regulated by Wnt pathway [32, 33]. This molecular mechanism of PKA-dependent *β*-catenin stabilization could be involved in ghrelin-induced *β*-catenin cytoplasmatic accumulation. Our data show that ghrelin enhances rOB viability through GHSR1a and PKA activation and, by acting on the same pathways, promotes stabilization of intracellular *β*-catenin levels. Therefore, we can speculate that the anabolic action of ghrelin in rOB is exerted through *β*-catenin stabilization. Further studies will be required to examine the direct involvement of *β*-catenin stabilization in ghrelin enhancement of rOB viability.

Loss- and gain-of-function mutations of *β*-catenin in osteoblasts affect not only bone formation but also bone resorption and osteoclast differentiation due to deregulation of the OPG gene. Activation of the Wnt/*β*-catenin pathway in osteoblasts suppresses bone resorption through upregulation of OPG expression and downregulation of RANKL expression [34, 35].

Our results show that ghrelin stimulates OPG expression and that pretreatment with the GHS-R1a antagonist, D-Lys^3^-GHRP-6, abolished the stimulatory action of ghrelin on OPG, thus indicating that ghrelin effects on OPG are specific and receptor mediated. The evidence that ghrelin induces OPG gene expression and reduces RANKL/OPG ratio suggests for the peptide an inhibitory role on osteoclastogenesis. This assumption is in line with the results obtained by exposing bone marrow precursor cells to the conditioned medium after 24 hr and 48 hr treatment with ghrelin. As expected, from the understanding of the OPG/RANK/RANKL system [36], we found that conditioned media from rOB treated with ghrelin inhibited, indeed, the development of TRAcP+ cells from bone marrow precursors. In Figure 7, panel A, typical multinucleated osteoclasts are outlined in the three experimental conditions and quantified in panel B.

It remains to be clarified whether ghrelin could have a direct action on osteoclastogenesis since the peptide is present in conditioned medium (CM) from rOB. This hypothesis is unlikely because CM from 24 h treated cells had no effect on multinucleated TRAcP positive cell formation. Consistent with this view is the here observed presence of the effect at 48 hours (Figure 7) that follows the earlier reduction of RANKL/OPG ratio at 24 h (Figure 6(c)), and the absence of a stimulatory effect by ghrelin on osteoclast differentiation observed by others in mouse bone marrow cultures [8].

Our data show a stimulatory action of ghrelin on OPG expression and consequently an inhibition of osteoblast mediated osteoclastogenesis and corroborate the results of Delhanty and colleagues [37] that demonstrated that osteoclastogenesis was faster in bone marrow cultures from ghrelin receptor deficient and ghrelin deficient mice and that ghrelin induced OPG expression in mouse osteoblastic cultures. Since D-Lys^3^-GHRP-6 abolished ghrelin effect on OPG expression in rOB, we can speculate that ghrelin affects indirectly osteoclastogenesis by binding GHS-R1a in primary rat osteoblasts. Our findings are in line with previous in vivo studies with hexarelin, a synthetic GH secretagogue that recognizes GHS-R1a. It has been reported, in fact, that long-term hexarelin administration inhibits bone resorption induced by gonadectomy in the rat [14]. Contrasting results have been reported on the effects of ghrelin on osteoclast activity. Costa and colleagues [8] have shown that ghrelin at a concentration of 10^−9 ^M increased the bone resorption activity of mature rat osteoclasts but did not modify osteoclast differentiation in mouse bone marrow cultures. However, the conditioned medium used in our experimental condition mimics the inhibitory effect of OPG addition in the osteoclastogenesis assay applied by Costa et al. [8]. More recently, van der Velde and colleagues [11] reported that ghrelin has a direct negative effect and indirect age-dependent positive action on osteoclastogenesis via a systemic pathway that is controlled by leptin. The importance of ghrelin in contrasting the “ageing” effect could be confirmed by our very preliminary data indicating that ghrelin (10^−8 ^M) induces a global hypermethylation of primary rat osteoblasts DNA (data not shown). This effect might counteract the typical global hypomethylation of DNA present in postmenopausal women with osteoporosis [38].

More research on this complex but fascinating area of gerontology and future in vitro and in vivo studies will be required to clarify the role of ghrelin in the regulation of bone turnover and to suggest the potential utility of GHS-R1a-agonists to stimulate bone formation and to inhibit osteoclastogenesis, thus improving bone structure in conditions such as osteoporosis.
